# Quantum pixel representations and compression for *N*-dimensional images

**DOI:** 10.1038/s41598-022-11024-y

**Published:** 2022-05-11

**Authors:** Mercy G. Amankwah, Daan Camps, E. Wes Bethel, Roel Van Beeumen, Talita Perciano

**Affiliations:** 1 Lawrence Berkeley National Laboratory, Computing Sciences Area, Berkeley, CA 94720 USA; 2 San Francisco State University, 1600 Holloway Avenue, San Francisco, CA 94132 USA; 3 Present Address: Department of Mathematics, Applied Mathematics and Statistics, Case Western Reserve University, Cleveland, OH USA

**Keywords:** Quantum information, Applied mathematics, Computer science, Scientific data, Software

## Abstract

We introduce a novel and uniform framework for quantum pixel representations that overarches many of the most popular representations proposed in the recent literature, such as (I)FRQI, (I)NEQR, MCRQI, and (I)NCQI. The proposed QPIXL framework results in more efficient circuit implementations and significantly reduces the gate complexity for all considered quantum pixel representations. Our method scales linearly in the number of pixels and does not use ancilla qubits. Furthermore, the circuits only consist of $R_y$ gates and $\text {CNOT}$ gates making them practical in the NISQ era. Additionally, we propose a circuit and image compression algorithm that is shown to be highly effective, being able to reduce the necessary gates to prepare an FRQI state for example scientific images by up to 90% without sacrificing image quality. Our algorithms are made publicly available as part of QPIXL++, a Quantum Image Pixel Library.

## Introduction

The growth in scientific data size and heterogeneity overwhelms current statistical and learning approaches for analysis and understanding. More specifically, the analysis of image-based data becomes increasingly challenging using current classical algorithms. Consequently, finding more efficient ways of handling scientific data is an important research priority.

Quantum computing holds the promise of speeding up computations in a wide variety of fields^1^, including image processing. One of the research challenges to make quantum computing a viable platform in the post-Moore era is to reduce the complexity of a quantum circuit to accommodate many qubits. The current and near-term quantum computers, known as noisy intermediate-scale quantum (NISQ) devices, are characterized by low qubit counts, high gate error rates, and suffer from short qubit decoherence times^2^. Hence, optimizing quantum circuits into short-depth circuits is extremely important to successfully produce high-fidelity results on NISQ devices.

Quantum image processing (QIMP) extends the classical image processing operations to the quantum computing framework^3^. QIMP algorithms are used on images that have been represented in a quantum state. A variety of quantum image representation (QIR) methods has been developed^4^. The flexible representation of quantum images (FRQI)^5,6^, the improved flexible representation of quantum images (IFRQI)^7^, the novel enhanced quantum representation (NEQR)^8^, the improved novel enhanced quantum representation (INEQR)^9^, the multi-channel representation of quantum images (MCRQI/MCQI)^10,11^, the novel quantum representation of color digital images (NCQI)^12^, and the improved novel quantum representation of color digital images (INCQI)^13^ are among the most powerful existing QIR methods. These QIR methods became extremely popular due to two main factors. First, their flexibility in encoding the positions and colors in a normalized quantum state. Second, image processing operations can be performed simultaneously on all pixels in the image by exploiting the superposition phenomenon of quantum mechanics.

In this paper, we introduce a uniform framework called the *quantum pixel representation* (QPIXL) that overarches all previously mentioned quantum image representations and probably many more. Furthermore, we propose a novel technique for preparing QPIXL representations that requires fewer quantum gates for all the different representations, compared to earlier results, and without introducing ancilla qubits. The proposed method makes use of an efficient synthesis technique for the uniformly controlled rotations^14^ and uses only $R_y$ gates and controlled-NOT ($\text {CNOT}$) gates, making the resulting circuits practical in the NISQ era. For example, the original FRQI state preparation method^5^ for an image with $N = 2^n$ grayscale pixels uses $n+1$ qubits in total, i.e., *n* qubits for encoding the position and 1 qubit for the color, and has a ${{\mathscr{O}}}(N^2)$ gate complexity. Recently, the FRQI gate complexity has been reduced to ${\mathscr{O}}(N \log _2N)$ at the price of introducing several extra ancilla qubits^7^. In contrast, our QPIXL method for preparing an FRQI state has only a gate complexity of ${\mathscr{O}}(N)$ and does not require extra ancilla qubits. Additionally, we introduce a compression strategy to further reduce the gate complexity of QPIXL representations. In our experiments, the compression algorithm allows us to further reduce the gate complexity by up to 90% without significantly sacrificing image quality. An implementation of our algorithms is publicly available as part of the Quantum Image Pixel Library (QPIXL++)^15^ at https://github.com/QuantumComputingLab. QPIXL++ is built based on QCLAB++^16,17^, which allows for creating and representing quantum circuits.

## Related work

Almost every image processing algorithm^18^ developed in the classical sense can also be developed in the quantum environment. These quantum versions may be computationally faster and may handle data more effectively by taking advantage of properties such as coherence, superposition, and entanglement associated with quantum science. How an image is represented on a quantum computer dramatically influences the image processing operations that can be applied. Hence, QIR has become a vital area of study in QIMP. Early approaches are the qubit lattice representation^19^ and the flexible representation of quantum images (FRQI)^5^. The latter, which is the FRQI method, forms the foundation of our work. The former is a quantum counterpart of classical image representation models without any significant performance improvement. At the same time, FRQI is based on quantum mechanical phenomena and captures both the color and geometry of an image in one quantum state. Besides its flexibility and the use of fewer qubits, FRQI can also perform both geometric and color operations on the image concurrently^20^.

Since the FRQI only uses one qubit for storing the color information, the number of measurements to accurately retrieve an image can be very large. The NEQR addresses this issue by storing the color information in orthogonal states allowing for color retrieval in a single measurement. Although the NEQR allows for accurate image retrieval, it requires significantly more qubits and does not utilize the superposition principle in the color qubit sequence, i.e., $\ell$ qubits basis states are used for images with bit depth $\ell$. On the other hand, the IFRQI combines both ideas and utilizes limited and discrete levels of superposition that are maximally distinguishable. The IFRQI, therefore, ensures accurate image retrieval with a small number of measurements; however, it requires $\log _2(N) - 2$ extra ancilla qubits. Other existing quantum image representation models are the quantum image representation for log-polar images (QUALPI)^21^, the *n*-qubit normal arbitrary superposition state (NASS)^22^, and the generalized quantum image representation (GQIR)^23^.

Several quantum image processing algorithms have been introduced in the literature using these QIRs. For example, Zhang et al.^24,25^ introduced an image edge extraction algorithm (QSobel) based on FRQI and also a quantum feature extraction framework based on NEQR. Jiang et al.^26^ recently proposed a new quantum image median filtering based on the NEQR. There are image segmentation algorithms that utilizes different QIRs along with the quantum Fourier transform^1,27^. Jiang et al.^23^ developed a new quantum image scaling up algorithm based on the GQIR. Li et al.^28^ developed a quantum version of the wavelet packet transforms based on the NASS. Zhou et al.^29^ proposed a quantum realization of the bilinear interpolation method for NEQR. There are several other examples in major application areas including image filtering^30–33^, image segmentation^34–36^, and machine learning^37–41^.

In order to run a quantum algorithm on a NISQ device, it first needs to be synthesized into elementary 1- and 2-qubit gates. The original implementation of the FRQI^5^ required ${\mathscr{O}}(N^2)$ elementary gates, while the more recent implementation by Khan^7^ reduced the complexity to ${\mathscr{O}}(64 N \log _2 N)$ elementary gates by introducing $\log _2(N) - 2$ extra ancilla qubits. We propose a novel QPIXL synthesis approach that reduces the FRQI gate complexity to ${\mathscr{O}}(2N)$, i.e., *N* rotation $R_y$ gates and *N* $\text {CNOT}$ gates, and does not require ancillary qubits. Furthermore, our QPIXL synthesis approach also reduces the original IFRQI gate complexity from ${\mathscr{O}}(p N \log _2 N)$ to only ${\mathscr{O}}(pN)$ and also gets rid of the ancilla qubits. Similar gains are obtained for preparing (I)NEQR, MCRQI, and (I)NCQI states.

## QPIXL: Quantum pixel representations

Some of the most widely used representations for quantum images, such as (I)FRQI^5,7^, (I)NEQR^8,9^, MCRQI^11^, and (I)NCQI^12,13^, can all be described by the following general definition for quantum image representations. This representation is similar to the pixel representation for images on traditional computers and captures both pixel colors and positions into a single quantum state $|I\rangle$ that we call a quantum pixel representation, QPIXL in short.

### Definition 1

(*Square QPIXL*) The quantum state for the QPIXL representation of a $2^m \times 2^m$ image $P = \left[ p_{ij}\right]$, where each pixel $p_{ij}$ has color $c_{ij}$, is given by the normalized state

1 $$\begin{aligned} |I\rangle = \frac{1}{2^m} \sum _{k=0}^{2^{2m}-1} |k\rangle \otimes |c_k\rangle , \end{aligned}$$

where $|k\rangle$ are the computational basis states on 2*m*-qubits and $|c_k\rangle$ is an encoding of the color information $c_{ij}$ in a quantum state on one or more qubits. The color values $|c_k\rangle$ should be regarded as a vectorized version of the 2D color values $c_{ij}$, i.e., $|c_k\rangle \mapsto c_{ij}$ for $k = i + j \cdot 2^m$.

We remark that the order of $|k\rangle$ and $|c_k\rangle$ in Definition 1 is reversed compared to the original definition^5,6^. Our ordering is consistent with the quantum circuit implementation for $|I\rangle$ provided in “QPIXL quantum circuit implementation” and in the original work^5,6^. Observe that the QPIXL state $|I\rangle$ creates an equal superposition over the computational basis states of the 2*m*-qubits in the first register, which encodes the pixel positions, and applies a tensor product with the state on the second register that encodes the color information. Definition 1 is general because it allows for flexibility in the type of color information and color encoding that is used. The mentioned QPIXL representations differ in their approach to map $c_{ij}$ to $|c_k\rangle$.

Since Definition 1 can trivially be extended to rectangular, 3D, and higher dimensional images, we will use the following more general definition.

### Definition 2

(*General QPIXL*) The quantum state for the QPIXL representation of an image of *N* pixels $p_k$ is given by the normalized quantum state

2 $$\begin{aligned} |I\rangle = \frac{1}{\sqrt{2^n}} \left( \sum _{k=0}^{N-1} |k\rangle \otimes |c_k\rangle + \sum _{k=N}^{2^n-1} |k\rangle \otimes |0\rangle \right) , \end{aligned}$$

where $n = \lceil {\log _2{N}\rceil }$, $|c_k\rangle$ is an encoding of the color information of pixel $p_k$, and $|k\rangle$ are the computational basis states on *n*-qubits.

Remark that in case the number of pixels *N* is not a power of 2, Definition 2 appends zero-valued pixels for $k = N,N+1,\ldots ,2^{\lceil {\log _2{N}}\rceil }-1$. Consequently, the state (2) is fully determined by the *N* pixel values $p_k$. Without loss of generality, we will assume that $N = 2^n$ in the remainder of the paper.

### QPIXL quantum circuit implementation

The preparation of a QPIXL state on a quantum computer can be considered as a state preparation procedure, i.e., $|I\rangle$ is the result of a quantum circuit $U_\text {QPIXL}$ applied to the all-zero state $|0\rangle ^{\otimes n+\ell }$, where *n* qubits are used to encode the pixel position and $\ell$ qubits are used for the color information.All QPIXL states are prepared in two steps: first creating an equal superposition over the *n* qubits that determine the pixel positions and afterwards adding the color information to the state by means of a unitary $U_{|c\rangle }$. In matrix notation, this procedure yields

3 $$\begin{aligned} |I\rangle = U_\text {QPIXL}|0\rangle ^{\otimes n+\ell } = U_{|c\rangle } (H^{\otimes n} \otimes I^{\otimes \ell } ) |0\rangle ^{\otimes n+\ell }, \end{aligned}$$

where $H^{\otimes n} \otimes I^{\otimes \ell }$ creates an equal superposition over the first *n* qubits:

4 $$\begin{aligned} ( H^{\otimes n} \otimes I^{\otimes \ell } ) |0\rangle ^{\otimes n+\ell } = H^{\otimes n} |0\rangle ^{\otimes n} \otimes |0\rangle ^{\otimes \ell } = \frac{1}{\sqrt{N}} \sum _{k=0}^{N-1} |k\rangle \otimes |0\rangle ^{\otimes \ell }. \end{aligned}$$

## FRQI in the QPIXL framework

The FRQI^5,6^ fits Definitions 1 and 2 of the QPIXL framework and is applicable to grayscale image data. An FRQI encoding uses only 1 qubit for the pixel intensity information $|c_k\rangle$. The color mapping used is bijective as discussed in detail by Li et al^22,42^. We define this mapping as follows.

### Definition 3

(*FRQI mapping*) For a grayscale image of *N* pixels $p_{k}$ where each pixel has a grayscale value $g_{k} \in \left[ 0, K\right]$, i.e., an integer value between 0 and the maximum intensity *K*, the QPIXL state with the FRQI mapping $|I_{\text {FRQI}}\rangle$ is defined by Definition 2 with the color mapping used in (2) given by^5,6,22,42^

5 $$\begin{aligned} |c_k\rangle&= \cos (\theta _k) |0\rangle + \sin (\theta _k)|1\rangle ,&\theta _k&= \frac{\pi /2}{K} \, g_k, \end{aligned}$$

with $$|0\rangle = \left[ {\begin{matrix} 1 \\ 0 \end{matrix}}\right]$$ and $$|1\rangle = \left[ {\begin{matrix} 0 \\ 1 \end{matrix}}\right]$$.

Observe that the FRQI representation of an *N*-pixel grayscale image requires $n +1$ qubits in total: *n* qubits for the pixel positions in $|k\rangle$ and 1 qubit for encoding the corresponding pixel intensity information in $|c_k\rangle$. By Eq. (5), we have that $\theta _k \in \left[ 0,\frac{\pi }{2}\right]$ and

6 $$\begin{aligned} |c_k\rangle = \begin{bmatrix} \cos (\theta _k) \\ \sin (\theta _k) \end{bmatrix}. \end{aligned}$$

Definition 3 is flexible because the grayscale value of each pixel $p_k$ can be encoded by choosing the angles $\theta _k$ accordingly. For example, consider an 8-bit grayscale image where each pixel $p_k$ has a grayscale value $g_k$ between 0 and 255, then the angles $\theta _k$ in Eq. (5) are given by^5,6,22,42^

7 $$\begin{aligned} \theta _k = \frac{\pi /2}{255} \, g_k. \end{aligned}$$

On the other hand, repeated measurement of the quantum state $|c_k\rangle$ yields the probabilities $\alpha _k^2 = \cos ^2(\theta _k)$ and $\beta _k^2 = \sin ^2(\theta _k)$ for the basis states $|0\rangle$ and $|1\rangle$, respectively. Hence, we can retrieve the grayscale values from these measurements by

8 $$\begin{aligned} g_k = \frac{255}{\pi /2} \arctan \left( \frac{\beta _k}{\alpha _k}\right) . \end{aligned}$$

We note that the color mapping defined in Eq. (5) has disadvantages when the images are transformed as discussed by Li et al^43^. In this case the authors propose extensions of the FRQI, named FRQIM and FRQIMC, in order to overcome the inconvenience to implement non-permutation transforms on FRQI. For the purposes of our work, we assume until “Other QPIXL mappings” that all image data is in grayscale and that we use the FRQI encoding from Definition 3.

### QPIXL-FRQI quantum circuit implementation

The circuit structure introduced in “QPIXL quantum circuit implementation” can be used to prepare the FRQI state on a quantum computer. In this case we have $\ell = 1$ and $U_{|c\rangle }$ that implements the mapping from Definition 3, we will denote this unitary as $U_{\mathscr{R}}$. This specification yields

9 $$\begin{aligned} |I_\text {FRQI}\rangle = \underbrace{U_{\mathscr{R}}(H^{\otimes n} \otimes I)}_{U_\text {FRQI}} |0\rangle ^{\otimes n+1}, \end{aligned}$$

with, according to Eq. (4),

10 $$\begin{aligned} (H^{\otimes n} \otimes I) |0\rangle ^{\otimes n+1}&= \frac{1}{\sqrt{N}} \sum _{k=0}^{N-1} |k\rangle \otimes |0\rangle = \frac{1}{\sqrt{N}} {\underbrace{\begin{bmatrix} 1&1&\cdots&1 \end{bmatrix}}_N}^\top \otimes \begin{bmatrix} 1&0 \end{bmatrix}^\top = \frac{1}{\sqrt{N}} {\underbrace{\begin{bmatrix} 1&0&1&0&\cdots&1&0 \end{bmatrix}}_{2N}}^\top . \end{aligned}$$

We define

11 $$\begin{aligned} U_{\mathscr{R}}&= R_y(2\theta _0) \oplus R_y(2\theta _1) \oplus \cdots \oplus R_y(2\theta _{N-1}) = \begin{bmatrix} R_y(2\theta _0) \\ &{} R_y(2\theta _1) \\ &{}&{} \ddots \\ &{}&{}&{} R_y(2\theta _{N-1}) \end{bmatrix}, \end{aligned}$$

with

12 $$\begin{aligned} R_y(2\theta _i) = \begin{bmatrix} \cos (\theta _i) &{} -\sin (\theta _i) \\ \sin (\theta _i) &{} \cos (\theta _i) \end{bmatrix}. \end{aligned}$$

Since $U_{\mathscr{R}}$ is by definition a block diagonal matrix with *N* $2 \times 2$ blocks and

13 $$\begin{aligned} R(2\theta _i) \begin{bmatrix} 1 \\ 0 \end{bmatrix} = \begin{bmatrix} \cos (\theta _i) \\ \sin (\theta _i) \end{bmatrix}, \end{aligned}$$

the prepared FRQI state (9) becomes

14 $$\begin{aligned} |I_\text {FRQI}\rangle&= \frac{1}{\sqrt{N}} \begin{bmatrix} \cos (\theta _0)&\sin (\theta _0)&\cos (\theta _1)&\sin (\theta _1)&\cdots&\cos (\theta _{N-1})&\sin (\theta _{N-1}) \end{bmatrix}^\top = \frac{1}{\sqrt{N}} \sum _{k=0}^{N-1} |k\rangle \otimes |c_k\rangle , \end{aligned}$$

which is a vector of length 2*N* holding the cosine and sine values of the angles of all the pixels. It can be directly verified that this definition of $U_{\mathscr{R}}$ agrees with Definition 3.

We can implement the $U_{\mathscr{R}}$ circuit on a quantum computer by using *N* multi-controlled $R_y$ gates^5^. We use the notation $C^n(R_y)$ for an $R_y$ gate with *n* control qubits. To illustrate this, we consider the FRQI encoding of a $2 \times 2$ image. This 4 pixels image can be implemented as follows using 3 qubits and 4 $C^2(R_y)$ gates:The angles $\theta _i$ correspond to the pixel values $p_i$ for $i = 0,1,2,3$ according to Eq. (5). The decomposition of the block diagonal matrix $U_{\mathscr{R}}$ into multi-controlled $R_y$ gates corresponds to the following matrix decomposition

$$\begin{aligned} \begin{bmatrix} I \\ &{} I \\ &{}&{} I \\ &{}&{}&{} R_y(2\theta _3) \end{bmatrix} \begin{bmatrix} I \\ &{} I \\ &{}&{} R_y(2\theta _2) \\ &{}&{}&{} I \end{bmatrix} \begin{bmatrix} I \\ &{} R_y(2\theta _1) \\ &{}&{} I \\ &{}&{}&{} I \end{bmatrix} \begin{bmatrix} R_y(2\theta _0) \\ &{} I \\ &{}&{} I \\ &{}&{}&{} I \end{bmatrix}, \end{aligned}$$

where each multi-controlled gate sets a single $2 \times 2$ block on the diagonal.

In order to actually run the $U_\text {FRQI}$ circuit on a quantum computer, we need to further synthesize the multi-controlled $R_y$ gates into elementary 1- and 2-qubit gates. For the case of $C^2(R_y)$ gates this can be done as follows^44^:yielding the following $U_\text {FRQI}$ circuit for the 4 pixels image example:By further decomposing the $C^1(R_y)$ gates into 3 $R_y$ and 2 CNOT gates as follows^1^,the directly implementable quantum circuit for $U_\text {FRQI}$ requires 44 single-qubit and 32 CNOT gates in total. In the general case for images with $N = 2^n$ pixels, every individual pixel value is encoded by a $C^n(R_y)$ gate. Decomposing these gates into 1- and 2-qubit gates by the method of Barenco et al.^44^ requires ${\mathscr{O}}(N)$ gates for every $C^n(R_y)$ gate. This results in an overall circuit complexity for $U_\text {FRQI}$ that scales quadratically in *N*, i.e., ${\mathscr{O}}(N^2)$ elementary gates are required to implement the full $U_\text {FRQI}$ circuit for an *N* pixels image on a quantum computer^5^. Khan^7^ recently improved the asymptotic complexity to ${\mathscr{O}}(N \log _2 N)$ by using $n - 2$ ancilla qubits.

## Optimal linear gate complexity

The complexity of implementing $U_\text {FRQI}$ is determined by the complexity of the circuit for $U_{\mathscr{R}}$, a block diagonal matrix with $2 \times 2$ blocks corresponding to the pixel values. In this section, we derive an alternative circuit implementation for $U_{\mathscr{R}}$ that requires quadratically fewer gates compared to the method proposed by Le et al.^5^, i.e., the asymptotic complexity of our novel implementation requires only ${\mathscr{O}}(N)$ quantum gates for a *N*-pixel image. Our new approach thus has optimal asymptotic scaling. It is also logarithmically faster compared to the method proposed by Khan^7^ and requires no ancilla qubits.

We start by reviewing a special case of the method introduced by Möttönen et al.^14^ to implement a block diagonal matrix in a quantum circuit. In that work, these circuits are called *uniformly controlled* $R_y$ *rotations* because they uniformly use all possible computational basis states in the control register. Let us define the nomenclature and diagrammatic notation for uniformly controlled $R_y$ rotations.

### Definition 4

(*Uniformly controlled* $R_y$ *rotations*) Given $\varvec{\theta }\in {\mathbb {R}}^N$, a vector of rotation angles, the uniformly controlled $R_y$ rotation is defined as

15 $$\begin{aligned} U_{\mathscr{R}}= R_y(\theta _0) \oplus R_y(\theta _1) \oplus \cdots \oplus R_y(\theta _{N-1}), \end{aligned}$$

and represented diagrammatically asThe dashed line indicates the $n \ = \log _2(N)$ qubits required for controlling the different diagonal positions in $U_{\mathscr{R}}$. The diagram on the right hand side uses a square control node to indicate that it is uniformly controlled by the first *n* qubits.

We know from the previous section that we can implement $U_{\mathscr{R}}$ by using *N* $C^n(R_y)$ gates. Here, we show that we can do this more efficiently by using a circuit that only consists of $R_y$ and $\text {CNOT}$ gates. As an illustrative example, let us consider the following circuit for 4 arbitrary angles ${\hat{\theta }}_0, \ldots , {\hat{\theta }}_3$:The following two properties of $R_y$ rotations are immediate:

$$\begin{aligned} R_y(\theta _0) \, R_y(\theta _1)&= R_y(\theta _0 + \theta _1), \\ X \, R_y(\theta ) \, X&= R_y(-\theta ), \end{aligned}$$

where *X* is a NOT gate $$\left[ {\begin{matrix} 0 &{} 1 \\ 1 &{} 0 \end{matrix}} \right]$$ that appears in the CNOT gates above. We can analyze the circuit above using these two simple properties and show that the circuit does create a block diagonal matrix with $2 \times 2$ blocks on the diagonal: the $R_y$ rotations on the 3rd qubit are all block diagonal matrices and the CNOT gates permute some of the blocks depending on the index of the first two control qubits. If we list the four $2 \times 2$ diagonal blocks in binary order, or equivalently the state of the 1st and 2nd qubit, we see that the circuit has the following effect on each block:

16 $$\begin{aligned} \begin{aligned} 00&:&\,R_y({\hat{\theta }}_3) \, R_y({\hat{\theta }}_2) \, R_y({\hat{\theta }}_1) \, R_y({\hat{\theta }}_0)&= R_y( \,{\hat{\theta }}_3 + {\hat{\theta }}_2 + {\hat{\theta }}_1 + {\hat{\theta }}_0),\\ 01&:&\, R_y({\hat{\theta }}_3) X R_y({\hat{\theta }}_2) \, R_y({\hat{\theta }}_1) X R_y({\hat{\theta }}_0)&= R_y( \,{\hat{\theta }}_3 - {\hat{\theta }}_2 - {\hat{\theta }}_1 + {\hat{\theta }}_0),\\ 10&:&X R_y({\hat{\theta }}_3) \, R_y({\hat{\theta }}_2) X R_y({\hat{\theta }}_1) \, R_y({\hat{\theta }}_0)&= R_y( - {\hat{\theta }}_3 - {\hat{\theta }}_2 + {\hat{\theta }}_1 + {\hat{\theta }}_0),\\ 11&:&X R_y({\hat{\theta }}_3) X R_y({\hat{\theta }}_2) X R_y({\hat{\theta }}_1) X R_y({\hat{\theta }}_0)&= R_y( - {\hat{\theta }}_3 + {\hat{\theta }}_2 - {\hat{\theta }}_1 + {\hat{\theta }}_0). \end{aligned} \end{aligned}$$

To implement a block diagonal matrix with this circuit, where the angles of the $R_y$ blocks correspond to $(\theta _0, \ldots , \theta _3)$, we get that the angles have to satisfy

$$\begin{aligned} \begin{bmatrix} \theta _0 \\ \theta _1 \\ \theta _2 \\ \theta _3 \end{bmatrix} = \begin{bmatrix} 1 &{} \,1 &{} \,1 &{} \,1 \\ 1 &{} -1 &{} -1 &{} \,1 \\ 1 &{} \,1 &{} -1 &{} -1 \\ 1 &{} -1 &{} \,1 &{} -1 \end{bmatrix} \begin{bmatrix} {\hat{\theta }}_0 \\ {\hat{\theta }}_1 \\ {\hat{\theta }}_2 \\ {\hat{\theta }}_3 \end{bmatrix}. \end{aligned}$$

This is a linear system with a specific structure, that we can rewrite as

17 $$\begin{aligned} \begin{bmatrix} \theta _0 \\ \theta _1 \\ \theta _2 \\ \theta _3 \end{bmatrix} = \begin{bmatrix} 1 &{} \,1 &{} \,1 &{} \,1 \\ 1 &{} -1 &{} \,1 &{} -1 \\ 1 &{} \,1 &{} -1 &{} -1 \\ 1 &{} -1 &{} -1 &{} \,1 \end{bmatrix} \begin{bmatrix} 1 &{} &{} &{} \\ &{} 1 &{} &{} \\ &{} &{} 0 &{} 1 \\ &{} &{} 1 &{} 0 \end{bmatrix} \begin{bmatrix} {\hat{\theta }}_0 \\ {\hat{\theta }}_1 \\ {\hat{\theta }}_2 \\ {\hat{\theta }}_3 \end{bmatrix} = ( {\hat{H}} \otimes {\hat{H}} ) P_G \begin{bmatrix} {\hat{\theta }}_0 \\ {\hat{\theta }}_1 \\ {\hat{\theta }}_2 \\ {\hat{\theta }}_3 \end{bmatrix}, \end{aligned}$$

where $${\hat{H}} = \left[ {\begin{matrix} 1 &{} 1 \\ 1 &{} -1 \end{matrix}}\right]$$ is a scaled version of the Hadamard gate and $P_G$ is the permutation matrix that transforms binary ordering to Gray code ordering.

It follows that, if we solve the linear system (17) for $({\hat{\theta }}_0, \ldots , {\hat{\theta }}_3)$, we can implement $U_{\mathscr{R}}$ for any $2 \times 2$ image with only 8 elementary gates: 4 $R_y$ rotations and 4 CNOT gates. The $U_{\mathscr{R}}$ circuit for the $2 \times 2$ example in the previous section required 74 gates: 42 1-qubit and 32 CNOT gates. Indeed, we have a quadratic improvement in gate complexity.

This strategy generalizes to block diagonal matrices $U_{\mathscr{R}}$ that have $2^n$ $R_y$ blocks on their diagonal^14^. The circuit structure consists of a sequence of length $2^n$ alternating between $R_y$ gates and CNOT gates. The $R_y$ gates act on the $(n+1)$st qubit, and thus correspond to block diagonal matrices with $2 \times 2$ blocks. The target qubit of the CNOT gates is set to the $(n+1)$st qubit and the control qubit for the $\ell$th CNOT gate is set to the bit where the $\ell$th and $(\ell + 1)$st Gray code differ. If $U_{\mathscr{R}}$ is determined by the angles $\varvec{\theta }= (\theta _0, \ldots , \theta _{2^n-1})$, the angles of the circuit ${\hat{\varvec{\theta }}} = ({\hat{\theta }}_0, \ldots , {\hat{\theta }}_{2^n-1})$ can be computed through the linear system:

18 $$\begin{aligned} \left( {\hat{H}}^{\otimes n} \, P_G \right) {\hat{\varvec{\theta }}} = \varvec{\theta }. \end{aligned}$$

As can be observed from the small-scale example (16), each angle ${\hat{\theta }}_i$ in the transformed domain contributes to every angle in $\varvec{\theta }$ in the original spatial domain. This means that there no longer exists a correspondence between an individual angle ${\hat{\theta }}_i$ and an individual pixel intensity $g_j$. As we will illustrate in “Experiments”, this can be considered an advantage as it allows one to approximate nonlocal correlations between pixels with fewer coefficients. In QPIXL++, Eq. (18) is solved with a matrix-free approach: the Gray permutation $P_G$ is performed in place and requires ${\mathscr{O}}(N)$ operations, the scaled Walsh–Hadamard transform ${\hat{H}}^{\otimes n}$ is implemented through a variant of the fast Walsh–Hadamard transform which requires ${\mathscr{O}}( N \log N)$ operations^45^. Pseudocode for both algorithms are provided in Algorithm 1 and Algorithm 2. Algorithm 2 lists a ${\mathscr{O}}(N)$ implementation for the Gray code permutation that requires a copy, while the QPIXL++ implementation achieves the same complexity without requiring a copy. Our implementation uses double precision arithmetic which suffices as the problem is well-conditioned, i.e., $\kappa ({\hat{H}}^{\otimes n} \, P_G) = 1$. 
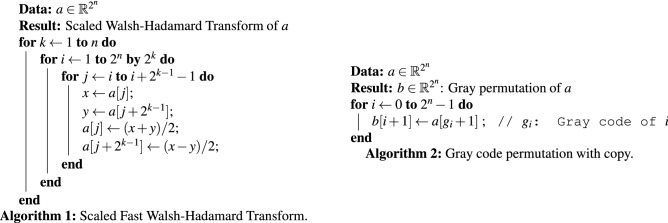

**Figure 1:**
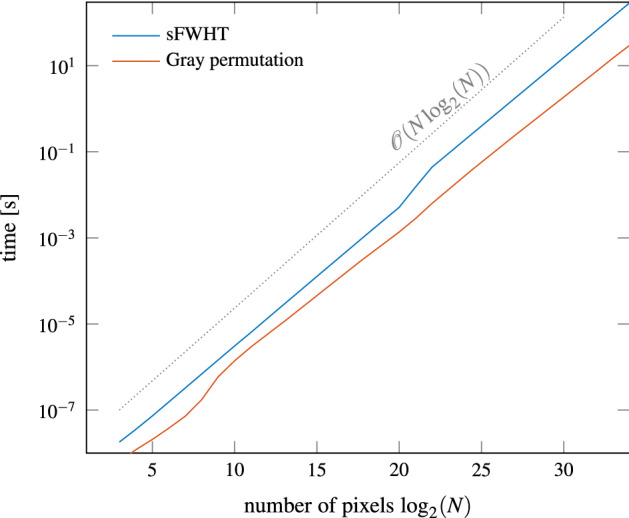
Scaling for scaled fast Walsh–Hadamard transform (sFWHT) and in-place Gray permutation with QPIXL++.

To show that our approach scales to large-scale images, we present benchmark data for solving the linear system (18) with the matrix-free methods that are implemented in QPIXL++. The results are shown in Figure 1 for randomly generated image data ranging from $2^3$ pixels up to $2^{34}$ pixels. The latter corresponds to the equivalent of an image with a resolution of more than 17 gigapixels, a 4K video fragment with 2070 frames, or a 1080p video fragment with 8285 frames. These timing results are obtained on a single core of an AMD Ryzen Threadripper 3990X 64-Core Processor @ 2.9 GHz with 256 GB RAM. Computing the coefficients for the data with $2^{34}$ pixels requires just over 5 min. This shows that our method easily scales to high resolution image and video data. The only current drawback is that this computation is memory bound due to the memory required to store the image data.

**Table 1:** Summary of gate complexities and qubit count for preparing the FRQI state $|I_\text {FRQI}\rangle$ for an image with $N = 2^n$ pixels with the approaches of Le et al.^5^ and Khan^7^ compared to our method.

FRQI	Gate complexity	Ancilla qubits	Total qubits
Le et al.^5^	${\mathscr{O}}(N^2)$	0	$n+1$
Khan^7^	${\mathscr{O}}(N \log _2 N)$	$n-2$	$2n-1$
QPIXL	${\mathscr{O}}(N)$	0	$n+1$

Our new $U_{\mathscr{R}}$ circuit requires only *N* $R_y$ rotation and *N* CNOT gates for an image with *N* pixels. As this scales linearly in the number of pixels, the asymptotic complexity of our approach is optimal. This is a quadratic improvement compared to the approach proposed by Le et al.^5^ that we described in “FRQI in the QPIXL framework”. The asymptotic complexities of both approaches are summarized in Table 1. We remark that as we require just 2 gates for every pixel, our constant prefactor is also considerably smaller compared to the works by Le et al.^5^ and Khan^7^.

## Compression

The proposed implementation of $U_{\mathscr{R}}$ as presented in “Optimal linear gate complexity” lends itself to an efficient circuit and thus image compression technique. As an example, we describe this idea for an FRQI image with 8 pixels.

**Figure 2:**
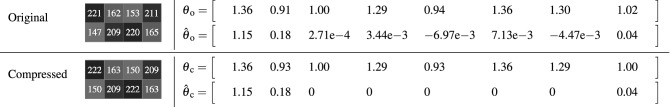
Compressing image data with 8 pixels arranged in a $2 \times 4$ grid.

Assume that the FRQI angle representation of an image is given by the vector $\varvec{\theta }\in {\mathbb {R}}^8$ and that we have computed the transformed vector ${\hat{\varvec{\theta }}} \in {\mathbb {R}}^8$ according to Eq. (18). The coefficients of ${\hat{\varvec{\theta }}}$ are then used in the following circuit for $U_{\mathscr{R}}$:For conciseness, we omit the $R_y$ labels and only state the rotation angle for the $R_y$ gates. Now assume that the image after the permuted Walsh-Hadamard transform is of the form ${\hat{\varvec{\theta }}} = ( {\hat{\theta }}_0, {\hat{\theta }}_1, \delta , \delta , \delta , \delta , \delta , {\hat{\theta }}_7)$, where $\delta$ are angles that can be considered negligible according to some compression criterion. A good approximation of the image is then given by ${\hat{\varvec{\theta }}} = ( {\hat{\theta }}_0, {\hat{\theta }}_1, 0, 0, 0, 0, 0, {\hat{\theta }}_7)$. This corresponds to the circuit below on the left where all $R_y$ rotations that have 0 angle after compression have been removed. This corresponds to a $62.5\%$ reduction in gates or compression level. This step results in a sequence of consecutive $\text {CNOT}$ gates all with the same target qubit and different control qubits. All these $\text {CNOT}$ gates commute with each other, so we can place them in arbitrary order. Furthermore, two consecutive $\text {CNOT}$ gates that have the same control qubit cancel each other since their product is the identity. The circuit below on the left has in the middle 1 CNOT with the first qubit as control, 2 $\text {CNOT}$s with the second qubit as control that cancel out, and 3 $\text {CNOT}$s with the third qubit as control of which two cancel with each other. It follows that the circuit on the left is equivalent to the circuit on the right with the redundant $\text {CNOT}$ gates removed.Figure 2 illustrates the compression algorithm for an actual image of 8 pixels where all transformed angles $\hat{\theta }_\text {o}$ below the tolerance $\delta = 0.01$ are set to zero. Note that, although the compression can influence all the angles $\theta _\text {c}$, the changes of the grayscale values are only in the range of $[-3,3]$. The reason for this is that Eq. (18) is well-conditioned so that small changes in $\hat{\theta }$ only lead to small changes in $\theta$ and its corresponding grayscale values.

As we describe next, this procedure easily generalizes to images of arbitrary size. After having computed ${\hat{\varvec{\theta }}}$, apply a compression criterion to set the negligible coefficients ${\hat{\theta }}_i$ to 0. Next, remove the corresponding $R_y$ rotations with 0 angle from the $U_{\mathscr{R}}$ circuit. Finally, perform a parity check on the control qubits of consecutive $\text {CNOT}$s in the $U_{\mathscr{R}}$ circuit: no $\text {CNOT}$ is required for control qubits with even parity, one $\text {CNOT}$ is required for control qubits with odd parity.

This algorithm is implemented in QPIXL++^15^. The compression criterion that we adopted selects a fixed percentage of the coefficients ${\hat{\theta }}_i$ with largest magnitude and thus of most importance. For example, a compression setting of $0\%$ retains all nonzero coefficients in ${\hat{\varvec{\theta }}}$, while a compression of $40\%$ sets the $40\%$ smallest coefficients $|{\hat{\theta }}_i|$ to zero. As we show in “Experiments”, this method can achieve high compression ratios while maintaining many features of the uncompressed image. The advantage of our approach is that we can discard coefficients after the Walsh-Hadamard transformation has been applied. In this way nonlocal correlations can be approximated with fewer coefficients compared to the untransformed data which can allow for improved compressibility. Furthermore, removing negligible angles in ${\hat{\varvec{\theta }}}$ is guaranteed to lead to small perturbations of the original angles $\varvec{\theta }$ as Eq. (18) is well-conditioned.

## Other QPIXL mappings

In this section, we extend our novel circuit implementation for $U_\text {FRQI}$ for grayscale data to different image representations that fit in Definitions 1 and 2. The key difference between all representations is the definition of the color encoding in the quantum state $|c_k\rangle$ from Definition 2. As long as we express this color mapping in terms of a combination of $R_y$ rotations, we can use our compressed implementation for the uniformly controlled $R_y$ rotations.

### IFRQI

The improved FRQI method introduced by Khan ^7^ combines ideas from the FRQI and NEQR representations. It improves upon the measurement problem for FRQI by allowing for only 4 discrete superpositions that are maximally distinguishable upon projective measurement in the computational basis. The IFRQI color mapping for a grayscale image with bit depth 2*p* is defined as follows.

#### Definition 5

(*IFRQI mapping*) For a grayscale image of *N* pixels where each pixel $p_k$ has a grayscale value $g_k \in [0, 2^{2p}-1]$ with binary representation $b^0_k b^1_k \cdots b^{2p-1}_k$, the IFRQI state $|I_\text {IFRQI}\rangle$ is defined by Definition 2 with the color mapping used in (2) given by

19 $$\begin{aligned} |c_k\rangle = |c_k^0 c_k^1 \cdots c_k^{p-1}\rangle , \end{aligned}$$

where, for $$i = 0, \ldots , p-1$$

$$\begin{aligned} |c_k^i\rangle&= \cos (\theta _k^i) |0\rangle + \sin (\theta _k^i)|1\rangle ,&\theta _k^i&= {\left\{ \begin{array}{ll} 0, &{} \text {if } b^{2i}_k b^{2i+1}_k = 00 \\ \frac{\pi }{5}, &{} \text {if } b^{2i}_k b^{2i+1}_k = 01 \\ \frac{\pi }{2} - \frac{\pi }{5}, &{} \text {if } b^{2i}_k b^{2i+1}_k = 10 \\ \frac{\pi }{2}, &{} \text {if } b^{2i}_k b^{2i+1}_k = 11 \end{array}\right. }. \end{aligned}$$

**Figure 3:**
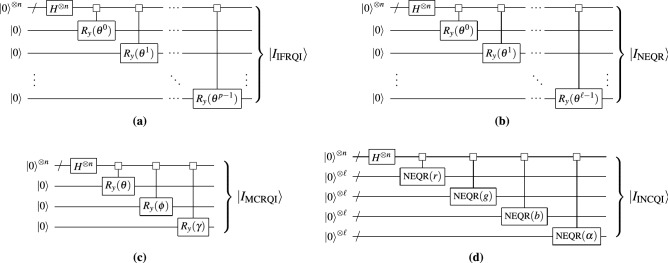
Circuits for the preparation of the IFRQI, NEQR, MCRQI, and INCQI states, where the uniformly controlled rotations can be compressed with our method.

We observe that the IFRQI mapping combines two bits of color information into one rotation. It follows that for an image with bit-depth 2*p*, we can prepare $|I_\text {IFRQI}\rangle$ using the circuit presented in Fig. 3a with *p* uniformly controlled $R_y$ rotations. The rotation angles $\varvec{\theta }^i$ correspond to bits 2*i* and $2i+1$ of all *N* pixels according to the values defined in Definition 5. These uniformly controlled rotations can be compressed independently with our compression algorithm. The gate and qubit complexites for IFRQI with our method compared to Khan ^7^ are listed in Table 2.

**Table 2:** Summary of gate complexities and qubit count for preparing the different QIR states covered in this paper and QPIXL for an image with $N = 2^n$ pixels.

Method	Literature				QPIXL	
	Reference	Gate complexity	Ancilla qubits	Total qubits	Gate complexity	Total qubits
FRQI	Le et al.^5^	${\mathscr{O}}(N^2)$	0	$n+1$	${\mathscr{O}}(N)$	$n+1$
	Khan^7^	${\mathscr{O}}(N \log _2 N)$	$n-2$	$2n-1$		
IFRQI	Khan^7^	${\mathscr{O}}(pN \log _2 N)$	$n-2$	$2n+p-2$	${\mathscr{O}}(pN)$	$n+p$
NEQR	Zhang et al.^8^	${\mathscr{O}}(\ell N \log _2 N)$	$n-2$	$2n+\ell -2$	${\mathscr{O}}(\ell N)$	$n+\ell$
INEQR	Jiang et al.^9^					
MCRQI	Sun et al.^10^	${\mathscr{O}}(3N^2)$	0	$n+3$	${\mathscr{O}}(3N)$	$n+3$
NCQI	Sang et al.^12^	${\mathscr{O}}(3\ell N \log _2N)$	$n-2$	$2n+3\ell -2$	${\mathscr{O}}(3\ell N)$	$n+3\ell$
INCQI	Su et al.^13^	${\mathscr{O}}(4\ell N \log _2N)$	$n-2$	$2n+4\ell -2$	${\mathscr{O}}(4\ell N)$	$n+4\ell$

For the IFRQI state, the bit depth is given by 2*p* and for the (I)NEQR, MCRQI, and (I)NCQI states the bit depth is given by $\ell$.

### NEQR

The idea for NEQR is to use a color mapping that directly encodes the length $\ell$ bitstring for the grayscale information in the computational basis states on $\ell$ qubits. The NEQR states for different colors are thus orthogonal and can be distinguished with a single projective measurement in the computational basis. In our QPIXL framework, the NEQR mapping can be defined as follows.

#### Definition 6

(*NEQR mapping*) For a grayscale image of *N* pixels where each pixel $p_k$ has a value $g_k \in [0, 2^{\ell }-1]$ with binary representation $b^0_k b^1_k \cdots b^{\ell -1}_k$, the NEQR state $|I_\text {NEQR}\rangle$ is defined by Definition 2 with the color mapping used in (2) given by

20 $$\begin{aligned} |c_k\rangle = |c_k^0 c_k^1 \cdots c_k^{\ell -1}\rangle , \end{aligned}$$

where

$$\begin{aligned} |c_k^i\rangle&= \cos (\theta _k^i) |0\rangle + \sin (\theta _k^i)|1\rangle ,&\theta _k^i&= {\left\{ \begin{array}{ll} 0, &{} \text {if } b^{i}_k = 0 \\ \frac{\pi }{2}, &{} \text {if } b^{i}_k = 1 \end{array}\right. }. \end{aligned}$$

By choosing the rotation angles $\theta ^i_k$ orthogonal, we ensure that the color information in $|I_\text {NEQR}\rangle$ can be retrieved through a single projective measurement. The NEQR state can be prepared through the circuit shown in Fig. 3b, where the uniformly controlled rotations can again be compressed with our method. The gate complexities for the uncompressed circuits are listed in Table 2.

### MCRQI

If we want to extend the applicability of the FRQI from grayscale to color image data, we have to allow for different color channels. This approach was dubbed multi-channel representation of quantum images (MCRQI)^11^. We adapt their definition for RGB image data to our formalism and make some minor modifications.

#### Definition 7

(*MCRQI mapping*) For a color image of *N* RGB pixels, where the color of each pixel $p_k$ is given by an RGB triplet $(r_k,g_k,b_k) \in \left[ 0, K\right]$, the MCRQI state $|I_{\text {MCRQI}}\rangle$ is defined by Definition 2 with the color mapping used in (2) given by

21 $$\begin{aligned} |c_k\rangle = |r_k g_k b_k\rangle , \end{aligned}$$

where

$$\begin{aligned} |r_k\rangle&= \cos (\theta _k) |0\rangle + \sin (\theta _k)|1\rangle ,&\theta _k&= \frac{\pi /2}{K} \, r_k, \\ |g_k\rangle&= \cos (\phi _k) |0\rangle + \sin (\phi _k)|1\rangle ,&\phi _k&= \frac{\pi /2}{K} \, g_k, \\ |b_k\rangle&= \cos (\gamma _k) |0\rangle + \sin (\gamma _k)|1\rangle ,&\gamma _k&= \frac{\pi /2}{K} \, b_k. \end{aligned}$$

We see that to encode the color information for an RGB image, we only require 2 additional qubits compared to grayscale data, which is a significant improvement over the classical case. Furthermore, we encode the color mapping as a tensor product of three qubit states, while Sun et al.^11^ encodes the information in the coefficients of the color qubits, which entangles their state. Our implementation has the advantage that the different color channels are easily treated separately, while the color information can still be retrieved thanks to the normalization constraint.

The circuit implementation of $|I_{\text {MCRQI}}\rangle$ for the RGB mapping defined in Definition 7 then simply combines three uniformly controlled rotation circuits with different target qubits and coefficient vectors determined by the respective color intensities as shown in Fig. 3c. As the RGB color channels are independent of each other and the uniformly controlled $R_y$ gates have different target qubits, each of them can be compressed separately. The asymptotic gate complexity of our method compared to the work by Sun et al.^11^ is listed in Table 2. As that work essentially uses the construction of Le et al.^5^, we obtain a quadratic improvement before compression.

### INCQI

Similarly to the NEQR, the (I)NCQI uses a color mapping directly encoding the length $\ell$ bitstring for each color value in a RGB$\alpha$ image in the computational basis stated on $\ell$ qbits. Consequently, this QIR can also be easily represented by our QPIXL framework through the mapping defined as follows.

#### Definition 8

(*INCQI mapping*) For a color image of N RGB$\alpha$ pixels, where the color of each pixel $p_k$ is given by a tuple $(r_k,g_k,b_k,\alpha _k)$ and each channel value in the range $[0, 2^{\ell }-1]$ has a binary representation, the INCQI state $|I_{\text {INCQI}}\rangle$ is defined by Definition 2 with the color mapping used in (2) given by

22 $$\begin{aligned} |c_k\rangle = |r_kg_kb_k\alpha _k\rangle = |r_k^0r_k^1\dots r_k^{\ell -1}g_k^0g_k^1\dots g_k^{\ell -1}b_k^0b_k^1\dots b_k^{\ell -1}\alpha _k^0\alpha _k^1\dots \alpha _k^{\ell -1}\rangle \end{aligned}$$

where

$$\begin{aligned} |r_k^i\rangle&= \cos (\theta _k^i) |0\rangle + \sin (\theta _k^i)|1\rangle ,&\theta _k^i&= {\left\{ \begin{array}{ll} 0, &{} \text {if } b^{i}_k = 0 \\ \frac{\pi }{2}, &{} \text {if } b^{i}_k = 1 \end{array}\right. }.\\ |g_k^i\rangle&= \cos (\phi _k^i) |0\rangle + \sin (\phi _k^i)|1\rangle ,&\phi _k^i&= {\left\{ \begin{array}{ll} 0, &{} \text {if } b^{i}_k = 0 \\ \frac{\pi }{2}, &{} \text {if } b^{i}_k = 1 \end{array}\right. }.\\ |b_k^i\rangle&= \cos (\gamma _k^i) |0\rangle + \sin (\gamma _k^i)|1\rangle ,&\gamma _k^i&= {\left\{ \begin{array}{ll} 0, &{} \text {if } b^{i}_k = 0 \\ \frac{\pi }{2}, &{} \text {if } b^{i}_k = 1 \end{array}\right. }.\\ |\alpha _k^i\rangle&= \cos (\psi ^i) |0\rangle + \sin (\psi _k^i)|1\rangle ,&\psi _k^i&= {\left\{ \begin{array}{ll} 0, &{} \text {if } b^{i}_k = 0 \\ \frac{\pi }{2}, &{} \text {if } b^{i}_k = 1 \end{array}\right. }. \end{aligned}$$

The definition above applies very similarly to the NCQI^12^, only removing channel $\alpha$ from the equation. The INCQI state can be prepared through the circuit shown in Fig. 3d. This circuit is built using an NEQR circuit for each channel of the ICNQI. Similarly to previous QIRs, the uniformly controlled rotations used here can also be compressed with our method. The gate complexities for the uncompressed circuits are listed in Table 2.

### Further extensions

We remark that multiple extensions and combinations of the ideas presented in this section are possible. For example, where MCRQI is a color version of FRQI and (I)NCQI is a color version of NEQR, we can similarly define a color version of IFRQI. We can also adapt IFRQI to group an arbitrary number of bits instead of the two bit pairing from Definition 5. This reduces the required number of qubits and gates at the cost of quantum states that are less distinguishable and thus require more measurements. It is even possible to use different QPIXL mappings for different RGB color channels. For example, we can use an FRQI mapping for the red channel, an IFRQI mapping for the green channel, and an NEQR mapping for the blue channel. Also, a generalized version of NEQR (GNEQR) was proposed by Li et al^46^, which is based on NEQR, INEQR, and NCQI. GNEQR uses $n+4\ell +2$ qubits to represent an image with $2^n$ pixels and bit depth of $\ell$ for 4 color channels. Using similar ideas described in this section, a QPIXL-based GNEQR would need $n+4\ell$ total number of qubits.

Finally, although we have presented this discussion for image data in an RGB($\alpha$) space, as in the work by Sun et al.^11^, our approach can be readily adapted to different color spaces and even multi-spectral or hyper-spectral data. In fact, different scientific applications frequently use images in different color spaces depending on the type of analysis needed. For example, the Y’CbCr space is known for its applicability to image compression. The I1I2I3 was created targeting specifically image segmentation. The HED space is advantageous in the medical field for the analysis of specific tissues. Similarly, multi-spectral and hyper-spectral data are used in areas such as geosciences and biology, for example, where experts acquire different satellite images and mass spectrometry images respectively. In all these cases, our general definition of quantum pixel representations can be directly applied.

## Experiments

This section describes a series of experiments that illustrate our proposed tools implemented in QPIXL++^15^. The current version of QPIXL++ supports the FRQI mapping from Definition 3 for grayscale image data of arbitrary dimensions.

**Figure 4:**
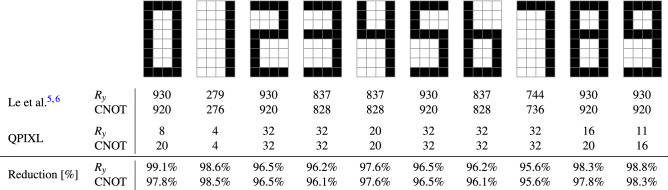
$8 \times 4$ image data containing digits 0–9, experiment replicated from Le et al.^6^. Gate complexities for the 6-qubit $U_{\mathscr{R}}$ circuits that prepare an exact representation of the image data. The last two rows provide the reduction in gate count for our method compared to Le et al.^5,6^. All circuits contain 5 Hadamard gates to create an equal superposition over the first register.

Our first experiment replicates a result from Le et al.^6^ with our $U_{\mathscr{R}}$ circuit and compares the gate complexities. In this test, we consider 10 images with an $8 \times 4$ resolution containing representations of the digits 0–9 as shown in Fig. 4. These binary images only contain black and white pixels. We require 5 qubits to encode the pixel location as we have 32 pixels in total.

The method of Le et al.^5^ requires one $C^5(R_y)$ gate for every pixel, bringing the total up to 32 $C^5(R_y)$ gates. Every $C^5(R_y)$ gate is further decomposed into 93 $R_y$ and 92 $\text {CNOT}$ gates. The experiment described by Le et al.^6^ reduces the number of $C^5(R_y)$ gates through a compression algorithm that groups pixels with the same grayscale value. This method is effective for the binary data in Fig. 4 as they report lossless compression ratios between $68.75\%$ and $90.63\%$. Figure 4 compares the number of 1-qubit $R_y$ and $\text {CNOT}$ gates for our method with the results from Le et al.^6^. We ran our compression algorithm with a compression level of 0% to the $U_{\mathscr{R}}$ circuit. Thus only coefficients in ${\hat{\varvec{\theta }}}$ that are exactly 0 are removed, which means that our circuits are exact. Figure 4 shows that our method always provides more than 95% reduction in gate count compared to the method from Le et al.^5,6^ for this example. The advantage of our method becomes even more outspoken for larger images due to the quadratic improvement.

**Figure 5:**
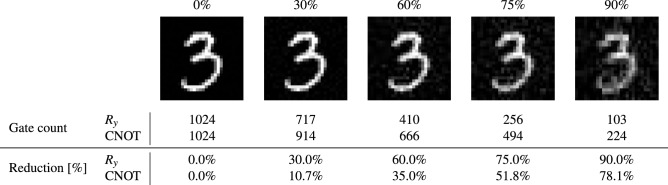
$28 \times 28$ image data from the MNIST^47,48^ database simulated with QPIXL++ at various compression levels and corresponding gate counts of the 11-qubit $U_{\mathscr{R}}$ circuit. The final two rows list the reduction in $R_y$ and $\text {CNOT}$ gates compared to the uncompressed circuits.

The next example we present concerns an image taken from the MNIST database^47,48^ of handwritten digits. The image of the digit “3” has a resolution of $28 \times 28$ pixels that is zero padded to an image with 1024 pixels in QPIXL++ which means that roughly 75% of the coefficients are used for the actual image data. Figure 5 shows the images that are simulated with QPIXL++ at 5 different compression levels. There are no visual artifacts at 30% compression and also the image at 60% compression is close to the original quality. The image with a 75% compression ratio has more visual artifacts but is still clearly recognizable, while at 90% compression the quality begins to drop significantly. The corresponding gate complexities for the $U_{\mathscr{R}}$ circuits are also listed in Fig. 5, all circuits contain 10 Hadamard gates to create the superposition in the first register. We observe that the reduction in $R_y$ gates is in perfect agreement with the compression ratio, but that there is generally a smaller reduction in $\text {CNOT}$ gates. This is in line with the expectations for our proposed compression algorithm described in “Compression”: not all $\text {CNOT}$ gates along a sequence of removable $R_y$ gates will cancel out. This experiment in particular clearly identifies a potential application of our QIR with compression to classification algorithms based on machine learning in quantum computers.

**Figure 6:**
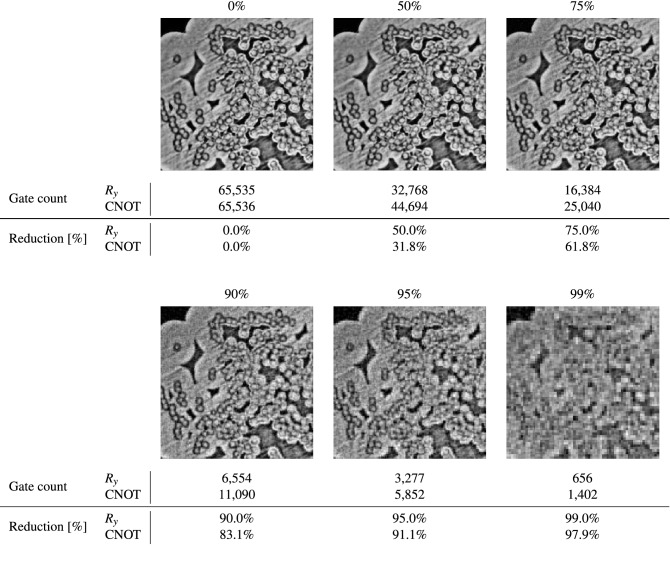
$256 \times 256$ image data from of a ceramic matrix composite sample^49^ acquired using microCT simulated with QPIXL++ at various compression levels and corresponding gate counts of the 17-qubit $U_{\mathscr{R}}$ circuit. The final two rows list the reduction in $R_y$ and $\text {CNOT}$ gates compared to the uncompressed circuits.

Our final example image stems from scientific data. This is a $256 \times 256$ pixels region from a cross-section of a ceramic matrix composite (fiber reinforced polymer)^49^ imaged with X-ray micro computed tomography (microCT) at the LBNL ALS beamline 8.3.2. This type of image is frequently acquired by material scientists to study the development of material deformation under stress. Consequently, image analysis algorithms to detect the circular patterns present in the image for example (cross-sections of fibers) become extremely important. As the dimensions of this grayscale image are already a power of 2, it does not need to be zero-padded. It contains both large scale structure and fine scale details. We require 16 qubits to encode the pixel locations and 1 for the grayscale intensities such that the $U_\text {FRQI}$ circuit has a total of 17 qubits. The uncompressed $U_{\mathscr{R}}$ circuit contains $2^{16}$ or 65,536 $\text {CNOT}$ and $R_y$ gates. We ran our compression algorithm on the data and the results are summarized in Fig. 6.

As can be observed, the compression algorithm is very effective for this image. Up to 75% compression can be achieved while still maintaining both the large scale structure and the finer details. The large scale structure is still preserved at 95% compression, but the acuteness in the finer details is lost at this compression level. It is only at 99% compression that the image becomes completely dominated by compression artifacts. It becomes clear from this last example that our compression approach becomes extremely interesting when analyzing scientific data: (1) the amount of data to be processed is reduced, and (2) the approach maintains details in the image necessary for further analysis, such as feature extraction for example.

## Conclusion

We have introduced an overarching framework for quantum pixel representations and showed how previously introduced image representations can be incorporated in the QPIXL framework. Among these methods are (I)FRQI, (I)NEQR, MCRQI, and (I)NCQI. We have proposed a novel circuit synthesis technique for preparing the quantum pixel representations on a quantum computer. This technique makes use of uniformly controlled $R_y$ rotations and significantly reduces the gate complexity for all aforementioned methods. Hence, the obtained circuits only require $R_y$ and $\text {CNOT}$ gates which makes them feasible for the NISQ era. Our method requires the solution of a particular linear system which can be solved classically in ${\mathscr{O}}(N \log N)$ time with a matrix-free approach. Furthermore, it allows for an efficient image compression algorithm that works on the transformed image data. Our experiments show that this compression approach is very effective for the FRQI mapping and can further reduce the number of gates by as much as 90% while still retaining the most prominent features of the image in the FRQI state. We repeatedly show how our method can have great impact on the analysis of scientific data and for quantum machine learning applications in the future. We have implemented and tested our algorithms in a publicly available software package QPIXL++^15^ which supports QASM output. Benchmark timings show that QPIXL++ has excellent scaling properties and can handle high resolution image and video data.

## Data Availability

The datasets analyzed during the current study are available in the QPIXL++ repository at https://github.com/QuantumComputingLab/qpixlpp.
