# Author Correction: Quantum pixel representations and compression for *N*-dimensional images

**DOI:** 10.1038/s41598-022-26352-2

**Published:** 2022-12-19

**Authors:** Mercy G. Amankwah, Daan Camps, E. Wes Bethel, Roel Van Beeumen, Talita Perciano

**Affiliations:** 1 Lawrence Berkeley National Laboratory, Computing Sciences Area, Berkeley, CA 94720 USA; 2 San Francisco State University, 1600 Holloway Avenue, San Francisco, CA 94132 USA; 3 Present Address: Department of Mathematics, Applied Mathematics and Statistics, Case Western Reserve University, Cleveland, OH USA

Correction to: *Scientific Reports* 10.1038/s41598-022-11024-y, published online 11 May 2022

The original version of this Article contained errors in the Figure legends of Figure 5 and Figure 6. The legends of these Figures were inadvertently switched.

The legend of Figure 5:

“$256 \times 256$ image data of a ceramic matrix composite sample^49^ acquired using microCT simulated with QPIXL++ at various compression levels and corresponding gate counts of the 17-qubit $U_{\mathscr{R}}$ circuit. The final two rows list the reduction in $R_y$ and $\text {CNOT}$ gates compared to the uncompressed circuits.”

now reads:

“$28 \times 28$ image data from the MNIST^47,48^ database simulated with QPIXL++ at various compression levels and corresponding gate counts of the 11-qubit $U_{\mathscr{R}}$ circuit. The final two rows list the reduction in $R_y$ and $\text {CNOT}$ gates compared to the uncompressed circuits.”

The legend of Figure 6:

“$28 \times 28$ image data from the MNIST^47,48^ database simulated with QPIXL++ at various compression levels and corresponding gate counts of the 11-qubit $U_{\mathscr{R}}$ circuit. The final two rows list the reduction in $R_y$ and $\text {CNOT}$ gates compared to the uncompressed circuits.”

now reads:

“$256 \times 256$ image data of a ceramic matrix composite sample^49^ acquired using microCT simulated with QPIXL++ at various compression levels and corresponding gate counts of the 17-qubit $U_{\mathscr{R}}$ circuit. The final two rows list the reduction in $R_y$ and $\text {CNOT}$ gates compared to the uncompressed circuits.”

The original Article has been corrected.

